# Measurement of mitochondrial respiration in permeabilized fish gills

**DOI:** 10.1242/jeb.216762

**Published:** 2020-02-19

**Authors:** Neal J. Dawson, Caroline Millet, Colin Selman, Neil B. Metcalfe

**Affiliations:** Institute of Biodiversity, Animal Health and Comparative Medicine, University of Glasgow, Glasgow G12 8QQ, UK

**Keywords:** LEAK, Oxidative phosphorylation, Mitochondrion-rich cells, Oxygraph, Oxygen consumption, Electron transport chain

## Abstract

Physiological investigations of fish gills have traditionally centred on the two principal functions of the gills: gas exchange and ion regulation. Mitochondrion-rich cells (MRCs) are primarily found within the gill filaments of fish, and are thought to proliferate in order to increase the ionoregulatory capacity of the gill in response to environmentally induced osmotic challenges. However, surprisingly little attention has been paid to the metabolic function of mitochondria within fish gills. Here, we describe and validate a simple protocol for the permeabilization of fish gills and subsequent measurement of mitochondrial respiration rates *in vitro*. Our protocol requires only small tissue samples (8 mg), exploits the natural structure of fish gills, does not require mechanical separation of the gill tissue (so is relatively quick to perform), and yields accurate and highly reproducible measurements of respiration rates. It offers great potential for the study of mitochondrial function in gills over a wide range of fish sizes and species.

## Abstract (precis)

**Summary:** Outline of a simple protocol for permeabilization of fish gills and measurement of mitochondrial physiology; this technique exploits the natural structure of gills and does not require mechanical separation.

## INTRODUCTION

It has been known for a long time that fish gills are highly metabolically active – indeed, Keys and Willmer (1932) reported the presence of mitochondrion-rich cells (MRCs) from fish gill epithelial tissue in their seminal studies of chloride excretory cells. Despite our increasing appreciation of the importance of fish gills not only for gas exchange but also for regulating the ionic and osmotic balance of fish through active secretory processes (Evans et al., 2005), the energetic costs and efficiency of gill tissue have remained relatively difficult to determine, primarily as a result of methodological constraints. Early methods of measuring mitochondrial properties in the gills of fish and mitochondrial respiration in the gills of bivalves relied on mitochondrial isolation from tissue homogenates (Galvez et al., 2002; Nogueira et al., 2013), but this is both time intensive and technically challenging (Hogeboom et al., 1948; Affourtit et al., 2012). Permeabilization of tissues by detergents has been suggested as a more physiologically relevant approach as it leaves the physical structure of mitochondria and other organelles intact, and eliminates any centrifugation bias present in traditional isolation techniques (Saks et al., 1998; Picard et al., 2011). Indeed, a study performed on oyster gills used traditional methods requiring the mechanical shredding of gill tissues followed by permeabilization with detergents during the run (Cahill et al., 2013). However, including the permeabilization step during the measurement of mitochondrial function leaves the possibility of over-permeabilization during prolonged runs, and the mechanical separation of tissue fibres required to facilitate permeabilization can also be time consuming and challenging to sustain sample-to-sample consistency (Larsen et al., 2014).

With these limitations in mind, we have developed a permeabilization technique to measure mitochondrial oxygen consumption rates in fish gills that is rapid and does not require any mechanical separation of the tissue, thus vastly increasing the accuracy and repeatability of measurements. Here, we describe and validate the approach using the gills of brown trout (*Salmo trutta*). The novel aspect of this method is the use of intact gill arches, with no mechanical manipulation of the gill filaments, exploiting the natural structure of gills to provide a large ratio of surface area to volume in order to facilitate uniform permeabilization by detergents. The simplicity of the protocol and small amount of tissue required should make it suitable for use in a wide range of fishes, so facilitating studies of the energetic cost of gill functions across diverse species and life stages.

## MATERIALS AND METHODS

### Animal collection and husbandry

One year old immature brown trout (*Salmo trutta* Linnaeus 1758; wet mass 8.04–72.74 g; standard length 98.9–183.0 mm; *n*=8) were purchased from a commercial hatchery (Northern Trout Ltd, Ae Fishery, Dumfries, UK), and subsequently housed at the University of Glasgow, where they were held in the laboratory for at least 4 weeks before experiments. Fish were housed in groups in 1 m diameter plastic tanks connected to a recirculation system supplied with dechlorinated tap water chilled to 12°C and were fed daily *ad libitum* with fish pellets [Micro LR 15P BST (25/100); EWOS, Bathgate, UK].

### Tissue and equipment preparation

**Fig. 1.**
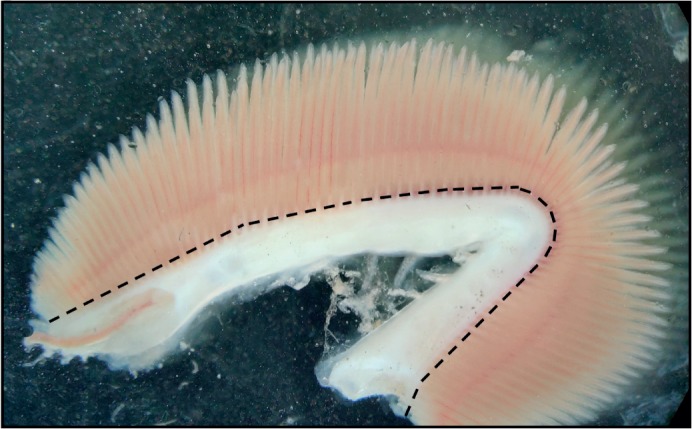
**Brown trout gill** **preparation.** The dashed line shows where saponin-permeabilized gills can be safely cut to avoid including connective tissue. Connective tissue adds to the mass of the sample but does not contribute to mitochondrial respiration; therefore, care should be taken to ensure it is properly dissected away from gill filaments.

Brown trout were killed by an overdose of benzocaine (1 g l^−1^ in 0.95% ethanol solution; project licence #P894B21.64) and were quickly weighed on an E2000D electronic balance (Sartorius, Göttingen, Germany). The gill arches were then excised within 2 min and transferred to ice-cold preservation buffer (in mmol l^−1^: 20 imidazole, 2.77 CaK_2_EGTA, 7.23 K_2_EGTA, 6.56 MgCl_2_, 20 taurine, 0.5 DTT, 50 potassium-methane sulfonate, 5.8 ATP and 15 creatine phosphate; pH 7.3). The gills, still attached to the arches, were then chemically permeabilized for 30 min in preservation buffer containing 50 μg ml^−1^ saponin. Saponin works in a concentration-dependent manner through interactions with cholesterol, which is found in high concentrations in the outer cell membrane and very low concentrations in the mitochondrial membrane, selectively removing it and creating holes predominately in the cholesterol-rich outer cell membrane (Jamur and Oliver, 2010). There was no effect on mitochondrial respiration rates when varying the permeabilization time between 15 and 45 min, which suggests that the permeabilization process is highly selective for the outer membrane, leaving the mitochondria intact. The gills and arches were then washed 3 times for 10 min in respiration buffer (in mmol l^−1^: 20 Hepes, 0.5 EGTA, 3 MgCl_2_, 60 potassium-lactobionate, 20 taurine, 10 KH_2_PO_4_ and 110 sucrose; with 1 mg ml^−1^ fatty acid-free bovine serum albumin, pH 7.3) to wash out endogenous molecules and remove excess saponin. The gills were blotted on dry wipes to remove excess buffer, cut away from the arches (see Fig. 1) and weighed on a Pioneer PA114C balance (Ohaus, Parsippany, NJ, USA) before respirometry measurements. It was critical to ensure that the gills were removed from the connective tissue closest to the arches, especially when using larger quantities of gill tissue, as the connective tissue could otherwise hit the polarographic oxygen sensor (POS) and/or stirrer, causing fluctuations in the O_2_ consumption signal. In order to prevent this, a small amount (∼0.1 mm) of gill tissue was left attached to the arches.

### Experimental design

*In situ* mitochondrial function was measured in 2 ml of respiration solution using a high-resolution respirometer (Oxygraph-2k with O2k-Fluorescence module; Oroboros Instruments, Innsbruck, Austria) at 12°C under continuous stirring. Gill fibres (8.0–24.9 mg wet mass) were allowed to sit for 5 min after being transferred to the chamber with the stirrer on. Respiration rate was measured from the rate of decline in O_2_ concentration within the chamber. In the first step, we added malate (2 mmol l^−1^) followed by pyruvate (5 mmol l^−1^) to stimulate LEAK or state 4 respiration (*L*_N_, LEAK respiration in the absence of ADP). ADP (5 mmol l^−1^) was then added to stimulate respiration via complex I (*P*_PM_, OXPHOS respiration with pyruvate and malate), reflecting the mitochondrial capacity for supporting oxidative phosphorylation (OXPHOS or state 3 respiration). Respiration was then measured after addition of glutamate (10 mmol l^−1^) (*P*_PMG_, OXPHOS respiration with pyruvate, malate and glutamate) and then succinate (25 mmol l^−1^) (*P*_PMGS_, OXPHOS respiration with pyruvate, malate, glutamate and succinate), to determine the maximal capacity for supporting OXPHOS via complex I and then complexes I+II (i.e. single and then convergent electron inputs to coenzyme Q), respectively. Cytochrome *c* (10 mmol l^−1^) was then added to assess the viability of our mitochondrial preparations (increases in respiration following cytochrome *c* additions are often used as an index of poor outer mitochondrial membrane integrity; Rasmussen and Rasmussen, 1997; Kuznetsov et al., 2004). To ensure O_2_ consumption rates were due to mitochondrial respiration and not to some other oxygen-consuming process, oligomycin [an *F*_0_*F*_1_ ATP synthase (ATPase) inhibitor] was added to inhibit respiration due to OXPHOS (so giving an alternative measure of LEAK respiration, *L*_Omy_); followed by antimycin A; antimycin is a coenzyme Q–cytochrome *c* reductase (complex III) inhibitor which prevents the flow of electrons through the electron transport chain, inhibiting remaining mitochondrial O_2_ consumption rates (state 5). Finally, ascorbate (0.5 mmol l^−1^) followed by *N*,*N*,*N*,*N*-tetramethyl-*p*-phenylenediamine (TMPD; 0.5 mmol l^−1^) was used to maximally stimulate complex IV (*P*_TM_, OXPHOS respiration with TMPD and ascorbate). Respiration rates were measured for at least 3 min in each condition until a steady state was reached, with rates expressed relative to the wet mass of gills. The reagents used in this section, and elsewhere throughout the Materials and Methods, were obtained from Sigma-Aldrich (Gillingham, Dorset, UK) unless otherwise stated.

The system was washed 3 times before and after each experimental run with both 70% ethanol and ddH_2_O. Calibration of the system was achieved by taking measurements of the background flux of the respiration buffer alone at 50, 100, 150, 200, 250, 300, 350, 400, 450, 500 and 550 nmol O_2_. We also explored respiration rates of gill tissues at the same O_2_ concentrations. At concentrations below 200 nmol O_2_, we observed decreases in gill O_2_ consumption rates; therefore, to ensure oxygen limitation was not a factor, experimental runs were conducted at O_2_ concentrations between 250 and 500 nmol O_2_. All experiments were run in duplicate.

### Gill tissue mitochondrial enzyme activity

The maximal activity (*V*_max_) of citrate synthase (CS) and cytochrome *c* oxidase (COX) is commonly used as a measure of mitochondrial density (Larsen et al., 2012), so would be expected to correlate with measurements of mitochondrial respiration rate per unit mass of tissue. Activity was assayed at 12°C as previously described (Du et al., 2017; Dawson et al., 2018) using a SpectraMaxPlus 384 spectrophotometer (Molecular Devices, Sunnyvale, CA, USA). Samples were homogenized in 20 volumes of ice-cold homogenization buffer [in mmol l^−1^: 100 KH_2_PO_4_, 1 EGTA, 1 EDTA and 1 phenylmethylsulfonyl fluoride (PMSF); pH 7.3]. Homogenates were then centrifuged at 1000 ***g*** at 4°C, and the supernatant collected. Enzyme activity was assayed in the following conditions (in mmol l^−1^): CS – 100 KH_2_PO_4_, 0.5 oxaloacetate, 0.15 acetyl-CoA, 0.15 5,5′-dithiobis-2-nitrobenzoic acid; pH 8.0; COX – 100 KH_2_PO_4_, 0.2 reduced cytochrome *c*; pH 7.3. *V*_max_ was measured in triplicate at 412 nm for CS (ε=14.15 mmol^−1^ l cm^−1^) and 550 nm for COX (ε=28.5 mmol^−1^ l cm^−1^). Enzyme activity is expressed in units of μmol substrate per gram of tissue per minute. Preliminary experiments determined that all substrate concentrations were saturating.

### Calculations and data analysis

All parameters were recorded continuously with DatLab 6.1.0.7 data acquisition software (Oroboros Instruments, Innsbruck, Austria). Respiration rates (pmol min^−1^ mg^−1^) were calculated by measuring the rate of oxygen consumption (pmol min^−1^) and dividing by the mass of gill tissue placed into the respirometer chamber (mg). Non-mitochondrial or background oxygen consumption (state 5) was subtracted from all other measurements. The quality of preparations was measured by examining the effect of exogenous cytochrome *c* on gill tissue respiration, and quantified by subtracting *P*_PMGS_ from the respiration rate measured after the addition of cytochrome *c* (*P*_PMGS_+cytochrome *c*) and dividing by *P*_PMGS_; when multiplied by 100, this gives the percentage increase in respiration after addition of cytochrome *c*, so that low values indicate a high-quality preparation. Two measures of the respiratory control ratio (RCR) were calculated by taking the ratio of respiration rate with pyruvate, malate and ADP (*P*_PM_) to that of each of the two LEAK respiration states (*L*_N_, *L*_Omy_).

Enzyme activity was calculated as in Eqn 1:(1)
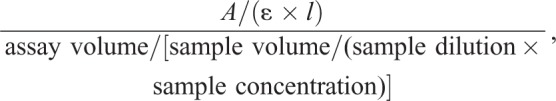
where *A* is the rate of change of absorbance over time (min^−1^), ε is the extinction coefficient (mmol^−1^ l cm^−1^) and *l* is path length (cm); assay volume and sample volume were measured in litres, and sample concentration in g l^−1^.

Data are presented as means±s.e.m. The repeatability between replicates was calculated as described in Lessells and Boag (1987). Results of linear regression analyses are presented as adjusted *R*^2^ and *P*-values. *P*<0.05 was considered significant.

## RESULTS AND DISCUSSION

**Fig. 2.**
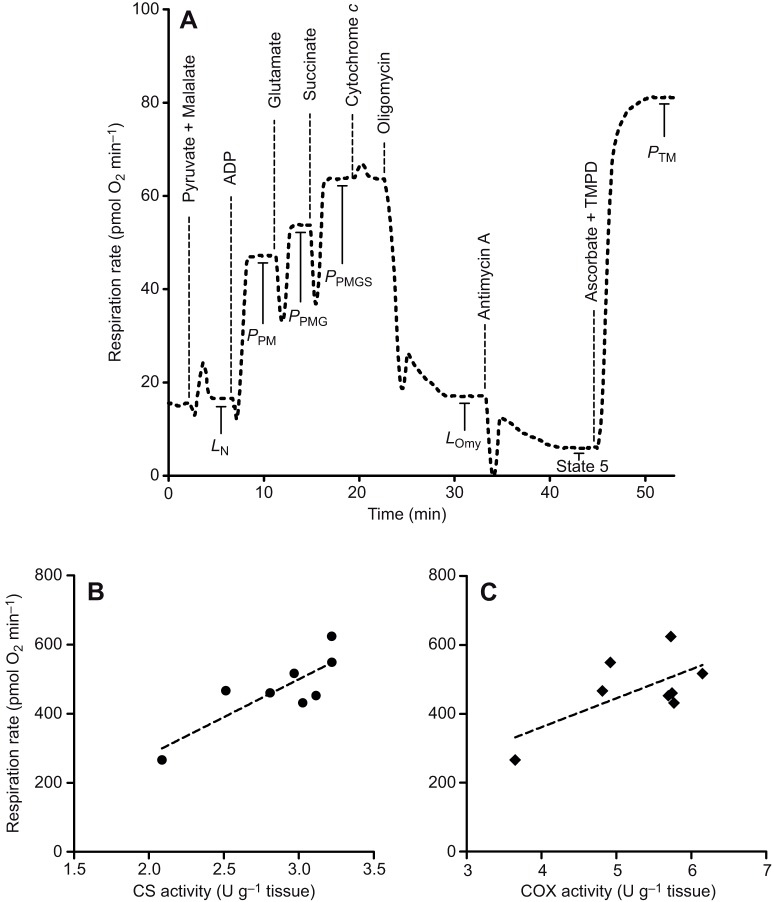
**An example trace of fish gill mitochondrial respiration and normalization to markers of mitochondrial volume.** (A) Representative experiment at 12°C on permeabilized gill fibres to measure mitochondrial respiration rate during oxidative phosphorylation. *L*_N_, LEAK respiration in the absence of ADP; *P*_PM_, oxidative phosphorylation (OXPHOS) with pyruvate and malate; *P*_PMG_, OXPHOS with pyruvate, malate and glutamate; *P*_PMGS_, OXPHOS with pyruvate, malate, glutamate and succinate; *L*_Omy_, LEAK respiration via oligomycin inhibition of ATP synthase; State 5, non-mitochondrial oxygen consumption; and *P*_TM_; complex IV respiration with TMPD and ascorbate as electron donors. (B,C) Relationship between maximal respiration rate (*P*_PMGS_) and either (B) citrate synthase (CS) activity or (C) cytochrome *c* oxidase (COX) activity, two common markers of mitochondrial abundance. Data points represent individual measurements and dashed lines represent linear regression lines. The slope for respiration rate versus CS activity was significantly different from zero (*R*^2^=0.689, *n*=8, *P*=0.011) while that for respiration rate versus COX activity was non-significant (*R*^2^=0.426, *n*=8, *P*=0.079).

The protocol described here successfully determined the mitochondrial respiration rate of permeabilized gill tissue from fish (Table 1, Fig. 2A). The respiration profile obtained using this technique demonstrated increases in O_2_ consumption rate at each step when the substrates pyruvate, malate, glutamate and succinate were sequentially added to stimulate electron transport chain components, and responded in an expected manner to the known mitochondrial complex inhibitors oligomycin and antimycin A (Fig. 2A). The addition of cytochrome *c* led to only a small percentage increase in respiration rate (Table 1), confirming that the preparations were of high quality. In comparison to semi-isolated, Dounce-homogenized, gill tissues (Braz-Mota et al., 2018), our preparation shows greater RCR values (7.4 versus 2.6–5.5), further confirming the high quality of mitochondrial preparations using this technique (Table 1). It is interesting to note that our respiration values are lower than those reported by Braz-Mota et al. (2018), although this is expected and has been shown in studies comparing different mitochondrial preparations in other taxa (Saks et al., 1998; Picard et al., 2011; Mahalingam et al., 2017). Mitochondrial respiration rates have classically been normalized using markers of mitochondrial volume, including CS and COX activity (Barrientos, 2002; Larsen et al., 2012; Dawson et al., 2018), and our mitochondrial respiration rates showed an excellent linear relationship (*P*=0.011, *R*^2^=0.689; Fig. 2B) with CS activity, suggesting that our measurements were the result of mitochondrion-based oxygen consumption. This is in line with previous experiments showing a strong correlation between mitochondrial content and CS activity (Larsen et al., 2012). While Larsen et al. (2012) also found a linear relationship between mitochondrial content and COX activity, this relationship was marginally non-significant in our study (*P*=0.079, *R*^2^=0.426; Fig. 2C), but a small sample size (*n*=8) meant that statistical power was low. Given the greater ease of measurement and stronger relationship with respiration rate, we suggest that CS is a more appropriate and more accessible method of normalizing gill mitochondrial respiration rates.

**Table 1.**
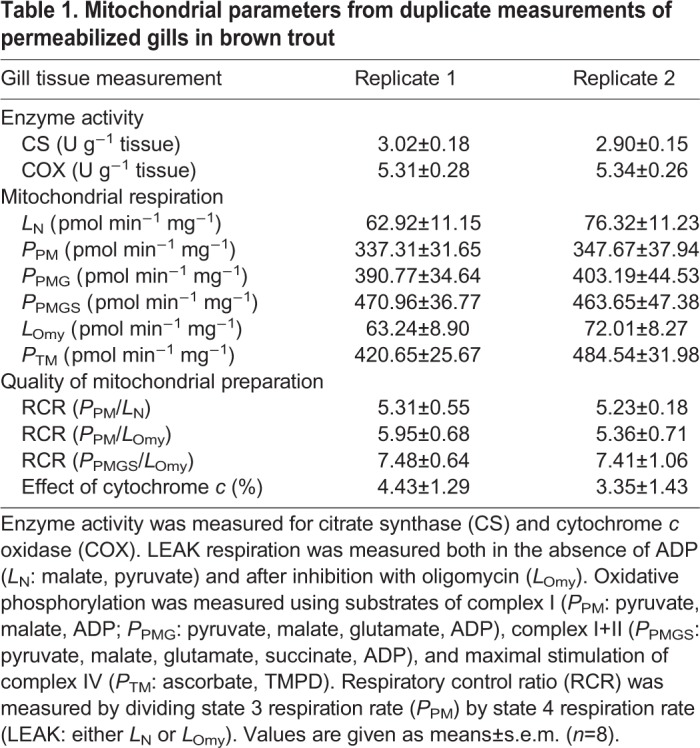
**Mitochondrial parameters from duplicate measurements of permeabilized gills in brown trout**

### Repeatability

Each measurement of gill mitochondrial respiration was run in duplicate in order to test the repeatability and accuracy of the technique. The two pieces of permeabilized gill tissue, sampled from a random location on a single gill arch, demonstrated remarkably similar respiration rates (Table 1; Table S1). In order to standardize our experimental design, our study did not explore the differences between anterior versus posterior gills. However, previous work shows that there may be morphological and functional differences between anterior and posterior gill arches (Huang et al., 2008) and this protocol could easily be adapted to test for similar changes in anterior versus posterior gill mitochondrial function. The average error between duplicate runs was 6.6% across all measured variables, suggesting a very high degree of repeatability between experimental runs (Table S1). It should be noted that the greatest variability was observed nearer to the end of the run, after the addition of the specific inhibitor oligomycin (12.2–13.2% difference between replicates), which has previously been demonstrated to be concentration dependent and variable in effectively inhibiting OXPHOS respiration in the presence of varying concentrations of ADP (Huijing and Slater, 1961; Masini et al., 1984; Ruas et al., 2016). In addition, the cumulative error of each addition of reagents may contribute to the increased error observed later in the run. Despite this, our results show that there was little variability between replicate measurements.

### Confirmation of mitochondrial respiration

Specific inhibitors of mitochondrial respiration were added in order to determine the contribution of non-mitochondrial respiration to the overall oxygen consumption rate. The two measures of LEAK (*L*_N_ and *L*_Omy_) were very similar (Table 1), indicating that *L*_N_ was not overestimated as a result of ATPase activity. The addition of antimycin A eliminated nearly all respiration, suggesting that the oxygen consumption rate observed in this experiment was indeed due to mitochondrial respiration (Fig. 2A).

### Scalability

The range of gill tissue mass investigated here (8.0–24.9 mg wet mass) encompassed the reported gill mass of many small aquatic fishes (Hughes, 1966); for example, the smallest amount of tissue that we used is well below the range of gill sizes for adult killifish, *Fundulus heteroclitus* (Karnaky et al., 1976). Even when using these small samples, the O_2_ consumption rate signal for OXPHOS was 5-fold larger than the LEAK respiration rate (RCR=5.51), suggesting that there was no loss of resolution. Therefore, it appears feasible that this technique could measure mitochondrial respiration rates in fishes with gills that are significantly smaller than those sizes studied here. We suggest that the protocol described here is scalable, and applicable to a wide range of fish species.

### Conclusion

We believe that measuring mitochondrial function in fish gills complements well-established techniques for exploring other aspects of gill function as it provides insight into the energetic cost of these other functions. Much attention has been given to how gills can be remodelled through changes in the number or quantity of MRCs in response to a variety of environmental factors including hypoxia, salinity and pharmaceuticals (Evans et al., 2005; Nilsson et al., 2012; Blair et al., 2016; Du et al., 2018). These changes would probably incur a large energetic cost as they involve rapid changes to the physical structure of the gills, with consequences for the way in which the gills supply ATP. There has also been much fervour surrounding the effects of gill morphology on ionoregulation, reviewed extensively in Hwang et al. (2011). It is clear that acid–base regulation as well as regulation of ions such as sodium, potassium and calcium require ATP in order to fuel the transmembrane transporters that support these processes (Hwang, et al., 2011; Lin and Randall, 1995). Some theories even suggest that the gills are required for ion regulation before they contribute appreciably to O_2_ uptake in developing fishes (Rombough, 2002). Perhaps changes in gill mitochondrial function will differ based on the ionoregulatory needs of the fish. Indeed, differences exist in the type and location of MRCs in freshwater fish (Chang et al., 2001), and there are two types of MRCs with high- and low-abundance Na^+^/K^+^-ATPases in freshwater rainbow trout (Galvez et al., 2002). It may be that more mitochondria, or more efficient mitochondria, are needed to support greater ATP demands required by increased ionoregulatory activity in fish gills. Do gills that undergo remarkable plastic remodelling require more mitochondrial ATP production? Can differences in gill mitochondrial efficiency explain interspecific variation in gill surface area, despite similar ionoregulatory needs? Do fish that transition from freshwater to seawater have more mitochondria, or more efficient mitochondria, to support the transition from ion uptake to secretion? Whatever the answers, the technique we describe here is particularly well suited to address these and other questions in greater detail than previously possible.

## Supplementary Material

Supplementary information
