# Supplementary material for: Measurement of mitochondrial respiration in permeabilized fish gills
Source: J Exp Biol. 2020 Feb 19;223(4):jeb216762. doi: 10.1242/jeb.216762 (PMC7044462; doi:10.1242/jeb.216762)
Supplement: Supplementary information [file jexbio-223-216762-s1.pdf]

Table S1. Variability between duplicate measurements and statistical results used in repeatability assessment of duplicate mitochondrial preparations. Repeatability was calculated as described in Lessells and Boag (1986).

Gill Tissue Measurement	% Difference	Sum of Squares Among Groups	Mean Squares Among Groups	Sum of Squares Within Groups	Mean Squares Within Groups	$F_{dfa^*,dfw^\dagger}$	Repeatability
Enzyme Activity							
Citrate Synthase (U*g tissue^{-1})	3.97 %	0.93	0.133	0.172	0.0215	$6.326_{7,8}$	0.607
Cytochrome c Oxidase (U*g tissue^{-1})	0.56 %	2.18	0.311	0.418	0.0522	$5.762_{7,8}$	0.618
Mitochondrial Respiration							
L_N (pmol*min^{-1}*mg^{-1})	7.56 %	7224	1032	7574.4	946.8	$1.090_{7,8}$	0.989
P_{PM} (pmol*min^{-1}*mg^{-1})	2.98 %	72128	10304	13832	1729	$5.751_{7,8}$	0.617
P_{PMG} (pmol*min^{-1}*mg^{-1})	3.08 %	119903	17129	22008	2751	$6.226_{7,8}$	0.605
P_{PMGS} (pmol*min^{-1}*mg^{-1})	1.55 %	161371	23053	50632	6329	$3.642_{7,8}$	0.752
L_{Omy} (pmol*min^{-1}*mg^{-1})	12.18 %	4865	608	1820	260	$0.428_{7,8}$	0.857
P_{TM} (pmol*min^{-1}*mg^{-1})	13.19 %	62048	8864	49704	6213	$1.427_{7,8}$	0.949
Quality of Mitochondrial Preparation							
RCR (P_{PM}/L_N)	1.51 %	13.265	1.895	9.008	1.126	$1.683_{7,8}$	0.921
RCR (P_{PM}/L_{Omy})	9.92 %	43.428	6.204	9.368	1.171	$5.299_{7,8}$	0.650
RCR (P_{PMGS}/L_{Omy})	0.85 %	33.313	4.759	17.760	2.220	$2.144_{7,8}$	0.875

**dfa* is the degrees of freedom amongst groups, †*dfw* is the degrees of freedom within groups
